# Age-related reductions in arousal-enhanced memory are moderated by trait emotion regulation

**DOI:** 10.1038/s41598-023-41741-x

**Published:** 2023-09-19

**Authors:** Kyoungeun Lee, Brialisse Sayre, Taylor A. James, Audrey Duarte

**Affiliations:** 1 School of Psychology, Georgia Institute of Technology, Atlanta, USA; 2 School of Psychology, University of Texas at Austin, Austin, USA; 3 School of Medicine, Mercer University, Macon, USA; 4 Department of Neurology, Emory University School of Medicine, Atlanta, USA

**Keywords:** Human behaviour, Cognitive ageing

## Abstract

Emotional arousal is known to enhance episodic memory in young adults. However, compared to valence, little is known about how healthy aging impacts arousal-enhanced memory effects. Furthermore, while emotion regulation is believed to improve with age, it is unclear how individual differences in emotion regulation influence arousal-enhanced memory. In this large-scale online study, we investigated the impact of age and individual differences in emotion regulation on arousal-enhanced memory. During encoding, participants made arousal ratings about negative, neutral, and positive images, and we compared their subsequent memory of high and low-arousal images. We found the impact of emotional arousal on memory was reduced with age, especially for older adults who habitually suppress their emotions. Our findings show that arousal-related memory benefits are reduced with advancing age, and that individual differences in habitual usage of emotion regulation impact these age-related alterations.

## Introduction

Many studies have investigated how emotion modulates memory and how the modulatory effect of emotion varies across the adult lifespan^1–5^. The majority of prior aging studies have focused on the impact of valence (positive or negative) on memory. An often-observed pattern shows that with older age, there is an increased memory preference for positive stimuli and/or a reduced memory preference for negative stimuli compared to neutral ones^5–7^. These positivity preferences have been tied to older adults’ superior emotion regulation ability compared to the younger adults^8,9^. An equally important dimension of emotion is arousal (calming or exciting/agitating), which is also a critical factor determining memory outcomes. Existing literature has consistently shown that arousal enhances memory, where stimuli inducing high states of arousal are remembered better than those inducing lower arousal—at least in younger adults^10–12^. Some studies have even proposed that the modulatory effect of arousal on memory may be greater than that of valence^13^.

However, it remains unclear whether the impact of arousal on memory differs across the adult lifespan. Some evidence shows comparable memory benefits for arousing compared to non-arousing stimuli for young and older adults^14–16^. These authors suggested that this preservation of arousal-induced memory benefits is due to the relative automaticity of processing highly arousing stimuli^17,18^—processes that are preserved in cognitively unimpaired aging^19,20^. However, other evidence shows reduced preference and/or increased avoidance of high-arousal materials in older adults compared to young, possibly due to age-related reductions in physiological flexibility e.g., Ref.^21^. In particular, it has been proposed that aging is associated with impaired homeostasis, resulting in older adults showing prolonged physiological arousal responses and experiencing delayed recovery from arousing events. The strength and vulnerability integration (SAVI) model^22^ posits that this diminished physiological flexibility hinders the effective regulation of high arousal in older adults, leading to more aversive response to high-arousal stimuli as they age. Thus, attentional and memory processing can be particularly compromised for high-arousal stimuli in older age^23–26^. For instance, Goot et al. found that high level of arousal in advertisement stimuli associated with worse memory retention for older adults, but not for younger adults^26^.

One potential factor that might contribute to these discrepant findings is that these studies categorized stimuli according to normative arousal ratings, which were primarily evaluated by younger adults. However, emerging evidence suggests that young and older adults rate arousal levels differently^23,24^. Specifically, older adults tend to evaluate negative stimuli as more arousing and positive stimuli as less arousing compared to young adults^23^. Thus, it is possible that pre-defined ‘low-arousing’ stimuli based on normative ratings in young adults might be perceived as more arousing for older adults, or pre-defined ‘high-arousing’ stimuli might be less arousing for older adults. To minimize the confounding effect of age-related differences in arousal rating, the current study utilized participants’ subjective arousal ratings rather than the normative ratings to classify high and low-arousing stimuli.

A number of previous studies have suggested that emotional regulation plays a crucial role in age-related changes in processing of emotional information (see Ref.^27^ for review). Consequently, we aimed to examine the potential influence of emotion regulation on the relationship between age and arousal-enhanced memory in this study. We focused on the two most commonly used and studied emotion regulation strategies: cognitive reappraisal and expressive suppression^28–30^. Cognitive reappraisal refers to re-evaluating or re-interpreting an emotional event to change its meaning or impact on one’s affective state^31,32^. Expressive suppression can be defined as inhibition or reduction of one’s behavioral response to emotional stimuli^31,32^. In general, reappraisal is thought to be a more effective strategy for regulating one’s emotion. According to the process model of emotion regulation, cognitive reappraisal occurs in the early phase of the emotion-generative process, consequently reducing the subjectively experienced emotion, physiological arousal, and behavioral expression^31,33^. Suppression, on the other hand, takes place in the late phase of emotion-generation—after the emotional response has been already induced, altering the external behavioral reactions (e.g., facial expression) and sometimes acting to heighten the physiological response to the emotional stimuli^31,33,34^. Older adults can often successfully regulate their emotions via reappraisal^34–36^, but may be less effective using suppression compared to the young, as evidenced by higher blood pressure and skin conductance^33,34^.

If high levels of arousal disrupt cognitive processing in older adults^24,25,36^, such as processes supporting event encoding, age-related reductions in arousal-enhanced memory may be most evident in older people who have a tendency to employ expressive suppression, compared to young people and those who employ reappraisal.

In this study, we measured habitual use of reappraisal and suppression using the Emotion Regulation Questionnaire (ERQ) to investigate whether individual differences in emotion regulation usage moderate age-related changes in arousal-enhanced memory. In order to mitigate the potential confounding effect of age-related difference in arousal ratings, we employed each participants' subjective arousal ratings instead of normative ratings to define the high and low arousing stimuli. Collectively, the main purpose of this study was to examine: (1) age-related differences in arousal-enhanced memory across the adult lifespan, and (2) the impact of individual differences in habitual emotion regulation on the relationship between age and arousal-enhanced memory. We hypothesized that the arousal-enhanced memory effects might be diminished with older age. We further predicted that the reduction would be most evident in individuals reporting high habitual suppression usage. To our knowledge, this is the first study exploring the relationship between habitual emotion regulation and arousal-enhanced memory across the adult lifespan. Although the primary focus of this study was the impact of arousal on memory, we additionally explored the effect of age and emotion regulation on positive and negative memory preferences. Given that positivity preferences have been attributed to older adult’s improved emotion regulation ability^8,9^, we predicted that greater positivity preferences and/or reduced negativity preferences might be most evident in older individuals with high habitual reappraisal usage.

## Methods

### Participants

Participants were recruited via Prolific (www.prolific.co), an online recruitment platform. Data collection was done from December 2020 to April 2021. Inclusion criteria were native English speakers located within the U.S., aged between 18 and 80. Of the 585 recruited participants, 520 completed the entire study procedure. Of the 520, 21 were excluded as they did not enter their subject IDs, making it impossible to link their encoding and retrieval data, and 52 were excluded due to poor quality responses (e.g., significant number of missing responses, identical responses for all or nearly all trials). In addition, due to server damage at the headquarters of Pavlovia (Ilixa Ltd., Nottinghamshire, UK, https://pavlovia.org/), the online experiment administration tool, 68 participants’ data were lost. Three participants were excluded from analyses due to outlier response bias values (*z*(hit rate) + *z*(false alarm rate))/2) on the memory retrieval task (± 3 S.D). Lastly, one participant was omitted for giving an age of zero. The remaining 375 participants (218 women, 146 men, 11 genderqueer or other; mean age: 41.05 ± 15.40, race/ethnicity: 232 non-Hispanic White, 40 Asian, 38 Black, 37 Hispanic/Latino, and 28 Other) were included in analyses. Informed consent was provided electronically, and participants were compensated at the rate of $10/h. This study was approved by the Georgia Institute of Technology Institutional Review Board. The study was carried out in accordance with the approved guidelines and regulations.

### Materials

#### Emotion regulation

The Emotion Regulation Questionnaire (ERQ^37^) was used to measure the habitual usage of two emotional regulation strategies: expressive suppression (ERQ-S) and cognitive reappraisal (ERQ-R). It is a 10-item self-report questionnaire, with four items measuring expressive suppression (e.g., “I control my emotions by not expressing them”) and six items measuring cognitive reappraisal (e.g., “I control my emotions by changing the way I think about the situation I’m in”). Respondents answered using a 7-point Likert scale from “strongly disagree” to “strongly agree.” The possible score of expressive suppression ranges 7–28 and that of cognitive reappraisal ranges 7–42. A high score indicates frequent use for the respective emotion regulation strategy.

#### Stimuli

For the emotional memory task, 396 images were selected from the International Affective Picture System (IAPS^38^) and Nencki Affective Picture System (NAPS^39^). Images were divided into three valence categories based on published standardized norms^38,39^ using the Self-Assessment Manikin (SAM) scale^40^ (valence: 1 = very negative, 9 = very positive); negative (1–4), neutral (4–6) and positive (6–9). We matched the normative arousal level of positive and negative images based on the published standardized norms^38,39^ using the 9-point SAM^40^ scale (arousal: 1 = relaxed, 9 = aroused). One hundred and thirty-two images were chosen for each valence category: positive (*M*_valence_ = 7.10 ± 0.38, *M*_arousal_ = 5.53 ± 0.56), neutral (*M*_valence_ = 5.59 ± 0.42, *M*_arousal_ = 4.09 ± 0.69), and negative (*M*_valence_ = 3.57 ± 0.83, *M*_arousal_ = 5.54 ± 0.38) valence categories. The normative arousal ratings for positive and negative images did not statistically differ (*p* = 0.77), while neutral images were less arousing than both positive and negative images (*p’*s < 0.001). Out of the 396 chosen images, 18 images were presented during the practice session. Then, 252 were presented during the encoding phase as "old" stimuli, and 126 were additionally presented during the retrieval phase as "new" stimuli. We counterbalanced the stimulus set, such that all stimuli served as both old and new stimuli across participants.

### Procedure

This study involved two experimental sessions. For the first session, participants performed the encoding phase of the emotional memory task and in the second session, they performed the retrieval phase. Before each phase, participants were guided by instructional videos and practice trials. Encoding and retrieval phases were separated by 48 h to reduce potential ceiling effects. The emotional memory task was conducted using Pavlovia (Ilixa Ltd., Nottinghamshire, UK, https://pavlovia.org/), an online experiment administration platform. A series of self-report questionnaires including the ERQ were completed in the first session before the encoding task. Depressive symptoms were measured with the Center for Epidemiological Studies Depression Scale Revised (CESD-R^41^). Since the current study was part of a larger research project, other self-report measures such as Pittsburg Sleep Quality Index (PSQI^42^) and Global Physical Activity Questionnaire (GPAQ^43^) were also collected. However, the measures that were not directly pertinent to the research questions of the current study were not considered in this paper. Demographic information was collected in the second session before the retrieval task. Self-report survey data were collected via Qualtrics (Qualtrics, Provo, UT), an online survey distribution tool.

### Emotional memory task

For the encoding phase, participants were asked to rate their level of arousal in response to each image. Participants were instructed to rate the intensity of the feeling they experienced when viewing each image by using 4-point scale: “not intense (1)”, “somewhat intense (2)”, “moderately intense (3)”, and “very intense (4).” The encoding phase was divided into four blocks, and each block included 63 trials with an equal number of positive, neutral and negative images. The trials were presented in a pseudorandom order. In total, 252 images were presented (84 positive, 84 neutral and 84 negative). Participants had up to 10 s to make a response. There was a 500 ms inter-trial interval period between trials.

Approximately 48 h later, participants performed the retrieval phase in the second session. During retrieval, all 252 old (i.e., shown during the encoding phase), and 126 new images were presented. The retrieval task was divided into six blocks. Each block included 63 trials (42 old, 21 new) with an equal number of positive, negative and neutral images. Within a block, trials were pseudo-randomly ordered. Participants were instructed to make recognition judgements for the presented images, and to indicate their degree of confidence associated with their decisions: “sure old,” “unsure old,” “unsure new,” “sure new.” For each trial, participants were given up to 10 s to make their response. There was a 500 ms inter-trial interval period between trials.

### Analyses

We estimated memory performance using the discrimination index, D-prime (*d′*; *z*(hit rate) − *z*(false alarm rate)), separately for positive, neutral and negative images. For positive and negative images, we further calculated *d’* according to subjective arousal ratings: high arousal (i.e., “moderately intense” or “very intense”) and low arousal (i.e., “not intense” or “somewhat intense”) for each subject. Given that neutral images are inherently low in arousal, we did not divide them by arousal rating. Trials responded to within 200 ms were considered invalid and were excluded from the analysis. Participants who had less than five valid trials in either the high or low arousal categories were not included in the calculation of *d*′ for that category (see Table 1 note; also see Supplementary Information for further detail).

**Table 1:** Memory discriminability (d′) and subjective arousal rating across participants.

	Positive	Neutral	Negative
Arousal rating	2.08 (0.55)	1.54 (0.41)	2.47 (0.48)
Overall *d*′	1.36 (0.65)	1.40 (0.71)	1.67 (0.72)
Low arousal *d*′	1.32 (0.65)^†^	–	1.53 (0.72)
High arousal *d*′	1.49 (0.75)^†^	–	1.87 (0.82)^†^

Entries are in the format: mean (S.D). Participants who had less than 5 valid trials in either the high or low arousal category were excluded in the calculation of d′ for that category ^†^*n* = Positive Low Intensity *d*′ (373), Positive High Intensity *d*′ (340), Negative High Intensity *d*′ (374).

We first examined how memory performance and average arousal ratings varied across valence categories. We further conducted Pearson correlations between all predictor and outcome variables to illustrate the general relationship among study variables. To investigate the influence of age and habitual emotion regulation on arousal-related memory enhancement—the primary research question of this study—hierarchical multiple regressions were conducted. In the first block, age and expressive suppression score (ERQ-S) were entered as predictors. In the second block, the interaction between age and suppression score was added. The outcome variable was the difference between high and low arousal memory (i.e., high arousal *d*′—low arousal *d*′). The analysis was done separately for positive and negative images. This regression model was repeated with reappraisal score (ERQ-R) in place of suppression score.

We conducted similar hierarchical regression analyses to investigate age-related differences in memory preferences for positive and negative, compared to neutral, stimuli. As for the above-described regression models, age and either suppression or reappraisal were entered in the first block, and the interaction term between them was added in the second block. The outcome variable was the memory preference to positive relative to neutral images (i.e., positive *d*′—neutral *d*′) and the memory preference to negative relative to neutral images (i.e., negative *d*′—neutral *d*′). Statistical analyses were performed in SPSS 27 (IBM Corp, Armonk, NY).

Gender, depressive symptom level (CESD-R score), and race/ethnicity were included as covariates. Gender (i.e., men, women and genderqueer/other) and race/ethnicity (i.e., non-Hispanic White, Racial/ethnic minority) were converted into dummy variables. The inclusion of these covariates did not significantly change the results of our main analyses. Therefore, we decided to report the statistical results from hierarchical regression models without these covariates. The results including the covariates are provided in the Supplementary Information section.

## Results

### Memory performance and subjective arousal rating

The means and standard deviations of subjective arousal ratings and memory discriminability for each valence type are shown in Table 1. First, we examined how subjective arousal ratings differed by valence. Results of a one-way ANOVA revealed a significant main effect of Valence [*F*(1.73,645.18) = 866.95, *p* < 0.001, *η*_*p*_^2^ = 0.70.] Arousal ratings were highest for negative images and lowest for neutral images (negative vs. positive: *t*(374) = 15.42, *p* < 0.001; negative vs. neutral: *t*(374) = 39.52, *p* < 0.001; positive vs. neutral: *t*(374) = 30.69, *p* < 0.001). We also ran a correlation analysis to determine whether age was related to subjective arousal ratings. We found no significant correlation between age and subjective arousal rating for positive or neutral images [*r’*s < 0.087, *p’*s > 0.05], while age was positively correlated with arousal rating for negative images [*r* = 0.144, *p* < 0.01; see Supplementary Fig. 1].

### Influence of age and emotional regulation on arousal-related memory enhancement

Descriptive statistics and correlation coefficients between predictor and outcome variables used in the hierarchical regressions are reported in Table 2. Correlations showed that older age was related to reduced usage of suppression, lower positive and negative *d*′, and decreased arousal-enhanced memory for negative images (see Supplementary Fig. 2).

**Table 2:** Descriptive statistics and correlation coefficient of study variables.

Variables (n)	Mean	SD	1	2	3	4	5	6
1. Age	41.05	15.40	–					
2. Suppression	14.24	6.09	− 0.227**					
3. Reappraisal	27.60	8.11	0.101	− 0.047	–			
4. Positive – Neutral *d*′	− 0.035	0.43	− 0.132*	− 0.003	0.026	–		
5. Negative – Neutral *d*′	0.27	0.44	− 0.141**	0.037	0.064	0.573**	–	
6. Arousal-enhanced Positive *d*′	0.18	0.42	− 0.097	0.003	− 0.026	− 0.004	0.020	–
7. Arousal-enhanced Negative *d*′	0.34	0.44	− 0.149**	− 0.013	− 0.067	0.007	0.146**	0.225**

*p < 0.05, **p < 0.01. The sample size (*n*) for each analysis was: 338 for Arousal-enhanced Positive *d*′, 374 for Arousal-enhanced Negative *d*′.

**Figure 1:**
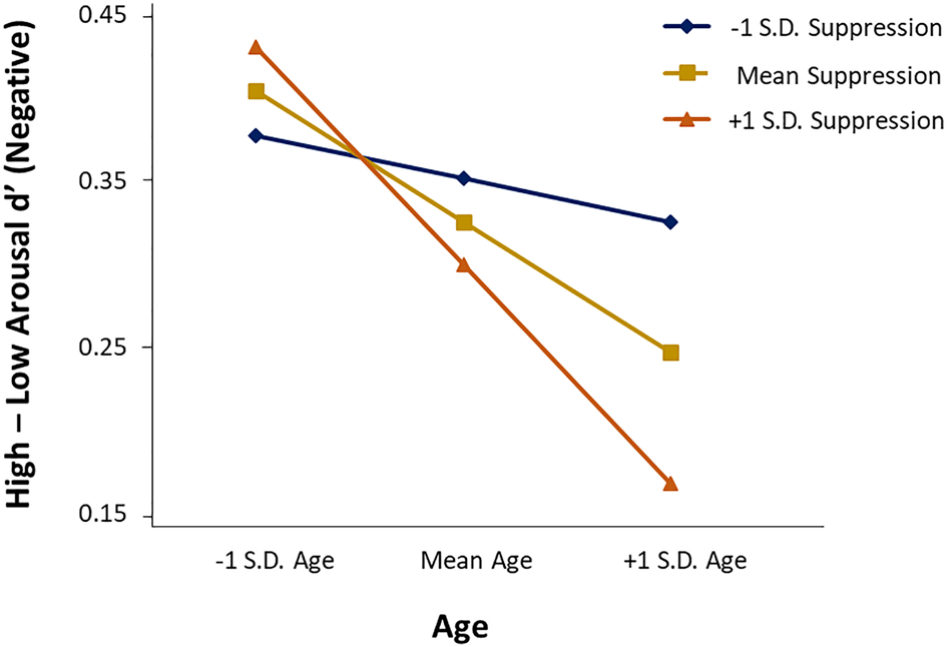
Age × suppression interaction on arousal-related memory benefit. This simple slopes plot shows the arousal-related memory benefit in function of age and suppression. This interaction shows that while age was associated with reduced arousal-related memory benefits for negative stimuli, the reduction was most pronounced in those with greater usage of habitual suppression.

The results of the hierarchical regressions predicting the primary outcome variable of interest in this study—the arousal-related memory benefit—are reported in the Table 3. The results showed that older age was related to reduced arousal-related memory benefits for negative but not positive images. In addition, expressive suppression scores significantly moderated the relationship between age and arousal-related memory enhancement for negative images. The simple slopes depicting this interaction are shown in Fig. 1. As can be seen in the figure, greater age was associated with a reduced arousal-induced memory benefit, particularly in people reporting more frequent usage of habitual suppression. No effects were significant for reappraisal scores.

**Table 3:** Hierarchical multiple regressions with age and emotion regulation predicting arousal-related memory benefits (High Arousal d′–Low Arousal d′).

	Model 1			Model 2		
	*B*	*SE B*	*β*	*B*	*SE B*	*β*
Positive (High arousal *d*′–Low arousal *d*′)						
Age	− 0.003	0.002	− 0.101	− 0.003	0.002	− 0.102
Suppression	− 0.001	0.004	− 0.019	0	0.008	0.007
Age × suppression	–	–	–	0	0	− 0.014
* R*^2^		0.010			0.010	
* F* for Δ*R*^2^		1.658			0.016	
Positive (High arousal *d*′–Low arousal *d*′)						
Age	0.003	0.002	− 0.096	− 0.003	0.002	− 0.092
Reappraisal	0.001	0.003	− 0.011	0.004	0.006	0.068
Age × reappraisal	–	–	–	0	0	− 0.092
*R*^2^		0.010			0.012	
*F* for Δ*R*^2^		1.621			0.738	
Negative (High arousal *d*′–Low arousal *d*′)						
Age	− 0.005	0.002	− 0.160**	− 0.005	0.002	− 0.178**
Suppression	− 0.004	0.004	− 0.049	0.010	0.007	0.140
Age × suppression	–	–	–	− 0.001	0	− 0.227*
* R*^2^		0.025			0.039	
* F* for Δ*R*^2^		4.685**			5.339*	
Negative (High arousal *d*′–Low arousal *d*′)						
Age	− 0.004	0.001	− 0.144**	− 0.004	0.001	− 0.141**
Reappraisal	− 0.003	0.003	− 0.052	0.005	0.004	− 0.098
Age × reappraisal	–	–	–	0	0	0.175
* R*^2^		0.025			0.033	
* F* for Δ*R*^2^		4.764**			3.115	

*p < 0.05, **p < 0.01; The sample size (*n*) for each analysis was: 338 for Positive High Arousal *d*′–Positive Low Arousal *d*′, 374 for Negative High Arousal *d*′–Negative Low Arousal *d*′.

### Influence of age and emotion regulation on positive and negative memory preferences

The results of the hierarchical regressions with outcome variables of memory preference for positive and negative images relative to neutral images are shown in Table 4. As can been seen in the table, age was significantly associated with a reduced preference for both positive and negative, relative to neutral, stimuli. Neither suppression nor reappraisal were related to these memory preferences.

**Table 4:** Hierarchical multiple regression with age and emotion regulation predicting positive and negative memory.

	Model 1			Model 2		
	*B*	*SE B*	*β*	*B*	*SE B*	*β*
Positive *d′*–Neutral *d′*						
Age	− 0.004	0.001	− 0.139**	− 0.004	0.001	− 0.140**
Suppression	− 0.002	0.004	− 0.034	− 0.002	0.007	0.026
Age × suppression	–	–	–	0	0	− 0.010
* R*^2^		0.018			0.018	
* F* for Δ*R*^2^		3.495*			0.011	
Negative *d′*–Neutral *d′*						
Age	− 0.004	0.002	− 0.139**	− 0.004	0.001	− 0.138**
Suppression	0	0.004	0.006	− 0.002	0.007	− 0.014
Age × suppression	–	–	–	0	0	− 0.024
* R*^2^		0.020			0.020	
* F* for Δ*R*^2^		3.756*			0.057	

*p < 0.05, ** *p* < 0.01; The regression results including reappraisal in place of suppression were similar, so they are not reported in the table.

## Discussion

In the present study, we explored how mnemonic benefits arising from experienced arousal are impacted by one’s age and habitual use of emotion regulation. Our results revealed that arousal-related memory benefits decreased with advancing age, exclusively for negative stimuli. Importantly, we found that the arousal-enhanced memory effect was most reduced in older individuals who reported the highest levels of habitual suppression. Habitual reappraisal was not significantly related to arousal-enhanced memory effects. Interestingly, current results did not support age-related positivity preferences. These results and their implications are discussed below.

### Age-related differences in subjective arousal ratings

Our finding shows that with increasing age, the arousal rating of negative stimuli, in particular, also increased. This somewhat aligns with previous research comparing older and younger adults' arousal ratings for emotional stimuli, which showed that older adults tend to rate negative stimuli as more arousing and positive stimuli as less arousing compared to younger adults^23,44–46^. Given these age-related differences in affective responses, it is advisable for future research in affective and cognitive aging to employ individual affect ratings of stimuli instead of relying on existing standardized ratings, especially those derived from young populations. This approach can contribute to a better understanding of age-related differences in emotional processing and foster more accurate interpretations of affective responses across different age groups.

### Age-related differences in arousal-related memory enhancement

We found that the arousal-related memory enhancement effect was reduced in older age, exclusively for the negative stimuli. This result is somewhat congruent with previous findings showing that cognitive processing can be disrupted by high experienced arousal, particularly in older age^24,25,36,47^. For example, older adults’ working memory is impaired when they experience high-arousal, both subjectively and physiologically^36^, and older adults’ episodic memory for emotional pictures is disproportionally compromised for highly-arousing negative stimuli, relative to low-arousing negative stimuli^24^. Collectively, these findings can be explained in the context of the strength and vulnerability integration model (SAVI^22^). This theoretical model proposes that older adults experience greater difficulty in emotion regulation when they are exposed to highly arousing information or contexts, due to their declining physiological flexibility to regulate heightened arousal states^22,48^. Therefore, older adults may be motivated to maintain low levels of emotional arousal^22^. Supporting this model, previous studies showed that older adults, compared to young, have an increased aversion to high-arousal contexts and stimuli^21,46^, and a greater preference for low-arousal stimuli^21,49^. Older adults might divert their attention away from highly arousing stimuli, in turn, reducing their encoding and subsequent memory for those stimuli. This idea is consistent with evidence showing that older adults rely on attentional diversion to regulate their emotions in daily life more than do younger adults^35,50^. Relatedly, some recent studies propose that the modulatory effect of arousal on selective attention changes with aging^51–54^. For instance, Gallant et al.^53^ demonstrated that in young adults, arousal promotes selectivity by eliciting significantly greater activation in brain regions specific to salient stimuli, as compared to non-salient stimuli. However, in older adults, high arousal relates to disrupted attentional selectivity, as evidenced by no difference in activation between salient and non-salient stimuli. Authors suggested that older adults may have difficulty selectively attending to salient information under high arousal conditions due to age-related changes in the noradrenergic modulatory system. Collectively, converging evidence has shown that there is an age-related decrease in the modulatory effect of arousal on attention and memory processing.

We found an age-related reduction in arousal-enhanced memory for negative, but not positive stimuli. It is worth noting that the impact of arousal (high–low *d′*) on memory was weaker for positive (Δ*d′* = 0.17) compared to negative (Δ*d′* = 0.33) stimuli in our study; a pattern similar to that reported in previous studies^24,55^. Consequently, it may have been difficult to detect any age-related reductions in arousal-induced memory benefits for positive events. It is also likely that older adults’ avoidance and/or aversive reaction to the arousing stimuli would be most evident for highly arousing negative stimuli.

### Age-related reductions in arousal-enhanced memory are most pronounced in habitual suppressors

Age-related reductions in arousal-enhanced memory were most pronounced in older adults with higher suppression scores. Older adults who habitually use suppression may experience a greater disruption in their ability to encode negative events when experiencing high levels of arousal. Prior studies have shown that using suppression results in a failure to reduce negative emotion and increased subjective and physiological arousal, especially for older adults^34,35,56^. Furthermore, suppression can be cognitively taxing since it involves continuous monitoring of one’s emotional response, leading to a reduction in available cognitive resources, such as those needed for episodic encoding^31,57^. Supporting this view, previous studies have reported impaired emotional memory when young individuals utilize suppression during encoding^28,31,58^. Collectively, these data demonstrate that suppression is not only affectively costly but also cognitively costly, especially in older age. It is possible that utilizing alternative emotion regulation strategies, such as acceptance-based approaches^59^ may be more beneficial in supporting both cognitive efficacy and emotional well-being. Consistent with this idea, the age-related reductions in arousal-enhanced memory were unaffected by habitual reappraisal.

### Reduced negative and positive memory preferences in older age

Somewhat surprisingly, our results showed that memory preferences for both positive and negative stimuli were reduced with age. There are several factors that might contribute to the lack of positivity preference in this study. First, some prior studies have suggested that the positivity effect is most evident in the oldest-old (e.g., age > 70; see Ref.^60^ for review). However, the mean age of the oldest third of our sample was relatively younger (age = 60.15) than other studies which have shown positivity effects (e.g., Ref.^61^, mean old age = 70.5; Ref.^62^, mean old age = 74.7). Relatedly, a meta-analysis reported that positivity effects are most evident in studies with greater age differences between older and younger adult groups^6^. However, the current study considered age as a continuous variable, and the age difference between the youngest third and oldest third of our sample was smaller (age difference = 36.3) than previous group comparison studies (e.g., Ref.^63^, age difference = 52.8; Ref.^64^, age difference = 48.3). Lastly, it has been suggested that depressive symptoms can affect older adults’ emotional preferences and well-being^3,65,66^. However, compared to typical older participants for lab-based studies, the online sample from the current study reported relatively higher scores of depressive symptoms. Specifically, the average depression score was higher in the oldest third of subjects (mean CESD-R = 8.71) than for older participants in lab-based studies showing age-related positivity preferences (e.g., Ref.^5^, CESD-R = 5.44; Ref.^8^, CESD-R = 7.09). Further studies examining positivity preferences in older adults need to consider various factors including age of older participants, mean age difference, and depressive symptoms.

### Limitations and future directions

The current study has some limitations that should be noted. First, although we found that age was related to reduced arousal-enhanced memory, which was impacted by habitual emotion regulation, we do not yet know the underlying mechanisms. It might be related to older adults’ diverted or impaired selective attention or their heightened efforts to downregulate the arousal response, but we need further empirical evidence before drawing these conclusions. Thus, future research should explicitly investigate underlying mechanisms by utilizing techniques such as eye-tracking, and neuroimaging. For instance, by employing eye-tracking during the encoding of high and low arousal stimuli, we can directly investigate whether older adults divert their attention away from highly arousing stimuli, while still attending to low arousing stimuli. Using fMRI, we could explore, for example, whether increased activity in brain regions responsible for downregulating arousal reactivity, such as the medial prefrontal cortex (mPFC) and amygdala^67^, respectively, could potentially be associated with less efficient encoding of highly arousing stimuli in older adults.

Second, as the current study was conducted online, we only focused on ‘subjective’ arousal and examined the modulatory effect of subjective arousal on memory. However, given that there are different types of arousal and various ways of inducing arousal, further lab-based study would be needed to investigate different types of arousal measures or arousal inducing methodologies. For example, physiological arousal measured by heart rate or skin conductance can be different from subjectively reported arousal. Previous studies have reported decreases in coherence between subjective arousal ratings and physiological measures of arousal in older compared to younger adults^55,68^. As such, the relationship between age and arousal-enhanced memory could vary based on the type of arousal measurement, given the greater disparity between subjective arousal and physiological arousal in older age. Also, rather than focusing on the arousal evoked by the stimuli, one could induce arousal by presenting additional exogenous stimuli or cues. For example, there are studies presenting films (e.g., comedy show) or sounds during or after the encoding of target stimuli^53,69^ in order to examine how ‘induced’ arousal affects encoding or post-encoding consolidation. Thus, future aging studies could consider physiological arousal and other arousal-inducing methodologies to examine the generalizability of the age-related reductions in arousal-enhanced memory effects.

## Conclusion

This study sheds light on the complex relationship between age, emotion regulation, and arousal-enhanced memory effects. Our findings indicate that the arousal-related mnemonic benefits decrease with advancing age, particularly for negative stimuli. The reduction in arousal-enhanced memory was most prominent in older adults, who habitually engage in emotional suppression. These results align with the SAVI model, suggesting that older adults may experience greater difficulty in cognitive processing when exposed to highly arousing information. In addition, these data highlight that utilization of emotional suppression can be both affectively and cognitively taxing, especially in older age. In conclusion, this study provides insights that it is important to consider the effect of arousal and habitual emotion regulation to understand the age-related changes in cognitive functions including episodic memory. Further investigations in this topic will be beneficial for developing targeted approaches to promote cognitive and emotional well-being across the adult lifespan.

### Supplementary Information

Supplementary Information.

## Data Availability

The annotated data is available at https://osf.io/3mhgn/.
