# Supplementary material for: Age-related reductions in arousal-enhanced memory are moderated by trait emotion regulation
Source: Sci Rep. 2023 Sep 19;13:15469. doi: 10.1038/s41598-023-41741-x (PMC10509193; doi:10.1038/s41598-023-41741-x)
Supplement: Supplementary file 1 — Supplementary Information. [file 41598_2023_41741_MOESM1_ESM.pdf]

Supplementary Table 1. *Hierarchical multiple regressions with covariates, age and emotion regulation predicting arousal-related memory benefits (High Arousal d′- Low Arousal d′)*

	Model 1			Model 2			Model 3		
	B	*SE B*	*β*	*B*	*SE B*	*β*	*B*	*SE B*	*β*
				Positive (High arousal *d′*- Low arousal *d′*)					
Gender (Men vs. Women)	-.041	.048	-.048	-.025	.049	-.029	-.025	.049	-.030
Gender (Men vs. Genderqueer)	.080	.136	.034	.060	.136	.025	.061	.137	.026
Race (White vs. non-White)	-.019	.047	-.022	-.056	.050	-.064	-.056	.051	-.065
CES-D	-.001	.002	-.044	-.002	.002	-.077	-.002	.002	-.078
Age	—	—	—	-.004	.002	-.133*	-.004	.002	-.135*
Suppression	—	—	—	.000	.004	-.007	.001	.008	.018
Age x Suppression	—	—	—	—	—	—	.000	.000	-.029
R^2		.005			.014			.014	
F for ΔR^2		.459			2.296			.071	
				Positive (High arousal *d′*- Low arousal *d′*)					
Gender (Men vs. Women)	-.041	.048	-.048	-.023	.048	-.027	-.022	.049	-.025
Gender (Men vs. Genderqueer)	.080	.136	.034	.059	.136	.025	.070	.136	.030
Race (White vs. non-White)	-.019	.047	-.022	-.055	.050	-.063	-.055	.050	-.063
CES-D	-.001	.002	-.044	-.003	.002	-.090	-.003	.002	-.090
Age	—	—	—	-.004	.002	-.130*	-.004	.002	-.126*
Reappraisal	—	—	—	-.002	.003	-.033	.003	.006	.049
Age x Reappraisal	—	—	—	—	—	—	.000	.000	-.095
R^2		.005			.020			.022	
F for ΔR^2		.459			2.448			.780	

				Negative (High arousal *d′*- Low arousal *d′*)					
Gender (Men vs. Women)	-.063	.048	-.070	-.058	.049	-.064	-.064	.048	-.071
Gender (Men vs. Genderqueer)	.027	.142	.010	-.007	.142	-.003	.001	.141	.000
Race (White vs. non-White)	.043	.047	.047	.010	.051	.011	.000	.050	.001
CES-D	.002	.002	.071	.002	.002	.051	.001	.002	.043
Age	—	—	—	-.004	.002	-.134*	-.004	.002	-.156**
Suppression	—	—	—	-.005	.004	-.070	.009	.007	.124
Age x Suppression	—	—	—	—	—	—	-.001	.000	-.232*
R^2		.014			.031			.045	
F for ΔR^2		1.299			3.149*			5.474*	
				Negative (High arousal *d′*- Low arousal *d′*)					
Gender (Men vs. Women)	-.063	.048	-.070	-.048	.048	-.053	-.044	.048	-.049
Gender (Men vs. Genderqueer)	.027	.142	.010	.006	.142	.002	.028	.142	.011
Race (White vs. non-White)	.043	.047	.047	.007	.051	.008	.006	.050	.007
CES-D	.002	.002	.071	.001	.002	.019	.001	.002	.020
Age	—	—	—	-.004	.002	-.126*	-.004	.002	-.123*
Reappraisal	—	—	—	-.003	.003	-.047	.006	.006	.104
Age x Reappraisal	—	—	—	—	—	—	.000	.000	-.175
R^2		.014			.028			.036	
F for ΔR^2		1.299			2.692			3.054	

Note: $^*p < 0.05$, $^{**}p < 0.01$; The sample size (*n*) for each analysis was: 338 for Positive High Arousal *d′* - Positive Low Arousal *d′*, 374 for Negative High Arousal *d′* -Negative Low Arousal *d′*.

Supplementary Table 2. *Hierarchical multiple regression with covariates, age and emotion regulation predicting positive and negative memory*

	Model 1			Model 2			Model 3		
	B	*SE B*	*β*	*B*	*SE B*	*β*	*B*	*SE B*	*β*
					Positive *d′*- Neutral *d′*				
Gender (Men vs. Women)	-.098	.046	-.112*	-.085	.047	-.097	-.085	.047	-.097
Gender (Men vs. Genderqueer)	-.078	.138	-.030	-.105	.138	-.041	-.104	.138	-.041
Race (White vs. non-White)	.049	.046	.055	.008	.049	.009	.007	.049	.008
CES-D	-.002	.002	-.055	-.003	.002	-.088	-.003	.002	-.089
Age	—	—	—	-.004	.002	-.151*	-.004	.002	-.153*
Suppression	—	—	—	-.002	.004	-.034	-.001	.007	-.013
Age x Suppression	—	—	—	—	—	—	.000	.000	-.025
R^2		.018			.036			.036	
F for ΔR^2		1.735			3.373*			.066	
					Negative *d′*- Neutral *d′*				
Gender (Men vs. Women)	-.052	.047	-.058	-.032	.048	-.036	-.031	.048	-.035
Gender (Men vs. Genderqueer)	-.010	.140	-.004	-.026	.140	-.010	-.026	.141	-.010
Race (White vs. non-White)	.086	.047	.095	.042	.050	.047	.043	.050	.047
CES-D	-.001	.002	-.040	-.003	.002	-.083	-.003	.002	-.082
Age	—	—	—	-.004	.002	-.142*	-.004	.002	-.140*
Suppression	—	—	—	.001	.004	.013	.000	.007	-.004
Age x Suppression	—	—	—	—	—	—	.000	.000	.020
R^2		.014			.030			.030	
F for ΔR^2		1.319			3.002			.039	

Note: $*p < 0.05$, $** p < 0.01$; The regression results including reappraisal in place of suppression were similar, so they are not reported in the table.

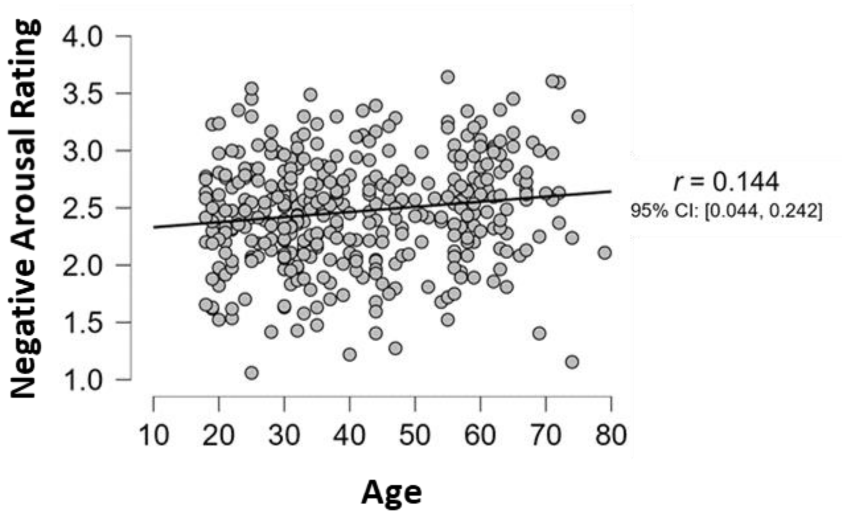

Supplementary Figure 1. Scatter plot showing the correlation between age and negative arousal rating.

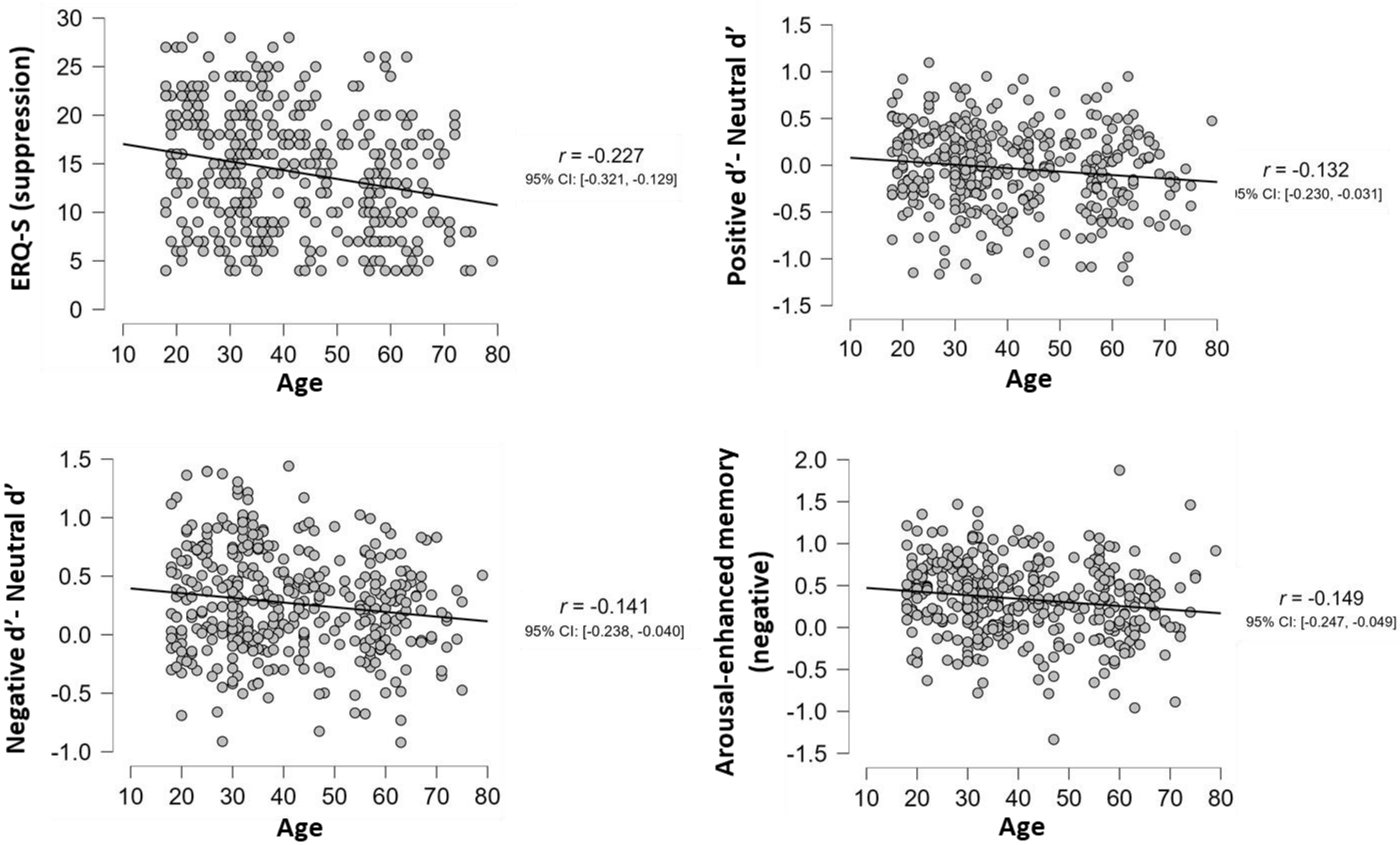

Supplementary Figure 2. Scatter plots showing the significant correlation between age and study variables.

Supplementary analyses examining the potential confounding effect of number of trials on observed outcome

In this study, the number of high and low arousal trials were not always balanced since we used each participant's subjective rating to define the high/low arousal condition (see supplementary Figure 3). We had 84 stimuli for each valence category (positive, neutral and negative), and we excluded participants who had less than 5 trials for either high or low arousal conditions from analysis. We acknowledge that 'less than 5 trials' is an arbitrary cut-off to estimate "meaningful" d′. To our knowledge, there is no consensus on the minimum number of trials to calculate d, so it is difficult to say what would be the optimal cut-off. Thus, to determine if the minimum number of trials may have an impact on our primary results, we conducted supplementary analyses.

First, we repeated our main analysis with a different cut-off value of minimum number of trials. By doing so, we aimed to determine if altering the cut-off decision had any impact on the observed results. If the changed decision of cut-off value makes no differences on the observed results we can conclude more confidently that our results were not biased by the number of trials. This time, we excluded participants who had less than 10 trials per condition. With this relatively conservative criterion, the results remained unchanged (see supplementary Table 3). Hence, we concluded that the decision of cut-off had not greatly impacted our findings. We kept using the original cut-off of 5 trials in the main manuscript to keep more participants in the analyses.

Second, we further examined whether there were any significant associations between the number of high/low arousal trials and d′and/or arousal-enhanced memory (i.e., high d′- low d′). If significant associations were found, it would suggest that the number of trials could indeed influence the memory outcomes, indicating that it should be considered as a confounding variable in our analysis. Conversely, if no significant associations were observed, it would imply that the number of trials might not have a substantial impact on our results. To validate these findings, we performed hierarchical regression analyses. Given that age is related with both the arousal ratings and d′, age was included as a covariate in the first block. Then, the number of high-arousal trials (or low-arousal trials) was added as a predictor in the second block. The outcome variable was either the high/low arousal d' or the difference between high and low arousal d′. As a result, we found that there was no significant influence of number of trials on d' or d' difference (see supplementary Table 4). We conclude that it is unlikely that the variability in the number of trials confounded our results.

Supplementary Table 3. Hierarchical multiple regressions with age and emotion regulation predicting arousal-related memory benefits (High Arousal d′- Low Arousal d′) with cut-off value of 10 trials

	Model 1			Model 2		
	B	*SE B*	*β*	*B*	*SE B*	*β*
			Positive (High arousal *d*′- Low arousal *d*′)			
Age	-.003	.002	-.134*	-.003	.002	-.137*
Suppression	-.002	.004	-.024	.004	.008	.068
Age x Suppression	—	—	—	0	0	-.052
R^2		.020			.021	
F for ΔR^2		3.009			.200	
			Positive (High arousal *d*′- Low arousal *d*′)			
Age	-.004	.001	-.144*	-.004	.001	-.140*
Reappraisal	.008	.006	-.028	.008	.006	.150
Age x Reappraisal	—	—	—	0	0	-.142
R^2		.020			.025	
F for ΔR^2		3.042*			1.577	
			Negative (High arousal *d*′- Low arousal *d*′)			
Age	-.004	.001	-.156**	-.005	.001	-.169**
Suppression	-.003	.004	-.047	.009	.007	.121
Age x Suppression	—	—	—	0	0	-.201*
R^2		.023			.034	
F for ΔR^2		4.304*			4.087*	
			Negative (High arousal *d*′- Low arousal *d*′)			
Age	-.004	.001	-.146**	-.004	.001	-.145**
Reappraisal	0	.003	-.002	0	.005	-.014
Age x Reappraisal	—	—	—	0	0	-.019
R^2		.021			.021	
F for ΔR^2		3.913*			0.033	

Note: *$p < 0.05$, ** $p < 0.01$; The sample size (*n*) for each analysis was: 299 for Positive High Arousal *d*′ - Positive Low Arousal *d*′, 363 for Negative High Arousal *d*′ -Negative Low Arousal *d*′.

Supplementary Table4. Relationship between number of trials and d′ and arousal-enhanced memory effect

	Model 1			Model 2		
	B	*SE B*	*β*	*B*	*SE B*	*β*
			Negative High arousal *d′*			
Age	-.010	.003	-.184**	-.009	.003	-.175**
Num of high arousal trials (negative)	—	—	—	-.004	.003	-.076
R^2		.034			.040	
F for ΔR^2		13.07**			2.22	
			Negative Low arousal *d′*			
Age	-.005	.002	-.113*	-.005	.002	-.111*
Num of high arousal trials (negative)	—	—	—	0	.002	-.017
R^2		.013			.013	
F for ΔR^2		4.80*			0.10	
			Negative (High arousal *d′*- Low arousal *d′*)			
Age	-.004	.001	-.149**	-.004	.001	-.138**
Num of high arousal trials (negative)	—	—	—	-.003	.001	-.095
R^2		.022			.031	
F for ΔR^2		8.49**			3.38	

Note: *p < 0.05, ** p < 0.01; We repeated the same analysis for positive stimuli, but there was no significant relationship between number of trials and d′ or d′ difference between high and low arousal conditions.

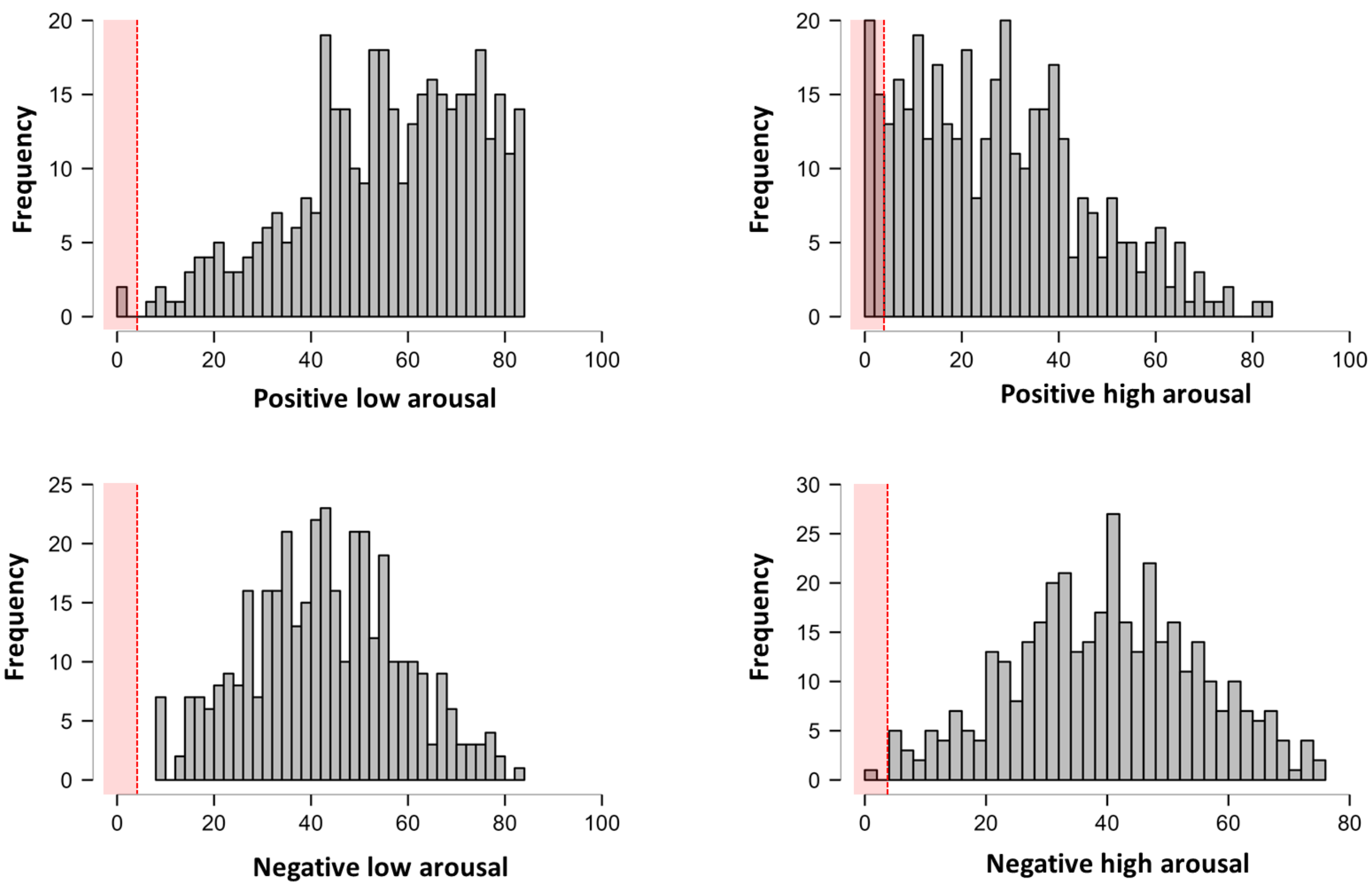

Supplementary Figure3. Histogram of number of trials for each valence and arousal conditions.

Note: These histograms illustrate the distribution of the number of trials for each valence and arousal condition across the participants. The red dashed line represents the cut-off value for the number of trials (e.g., fewer than 5 trials). Participants with less than 5 trials, denoted by the red box in the figure, were excluded from the main analysis.
